# Supplementary material for: A Humanized Yeast Phenomic Model of Deoxycytidine Kinase to Predict Genetic Buffering of Nucleoside Analog Cytotoxicity
Source: Genes (Basel). 2019 Sep 30;10(10):770. doi: 10.3390/genes10100770 (PMC6826991; doi:10.3390/genes10100770)
Supplement: Supplementary file 1 [file genes-10-00770-s001.zip › Additional_Files/Additional_File7_GOTermHeatmaps.zip!/Additional_File7_GOTermHeatmaps/transcription_factor_activity,_RNA_polymerase_II_transcription_factor_binding.pdf]

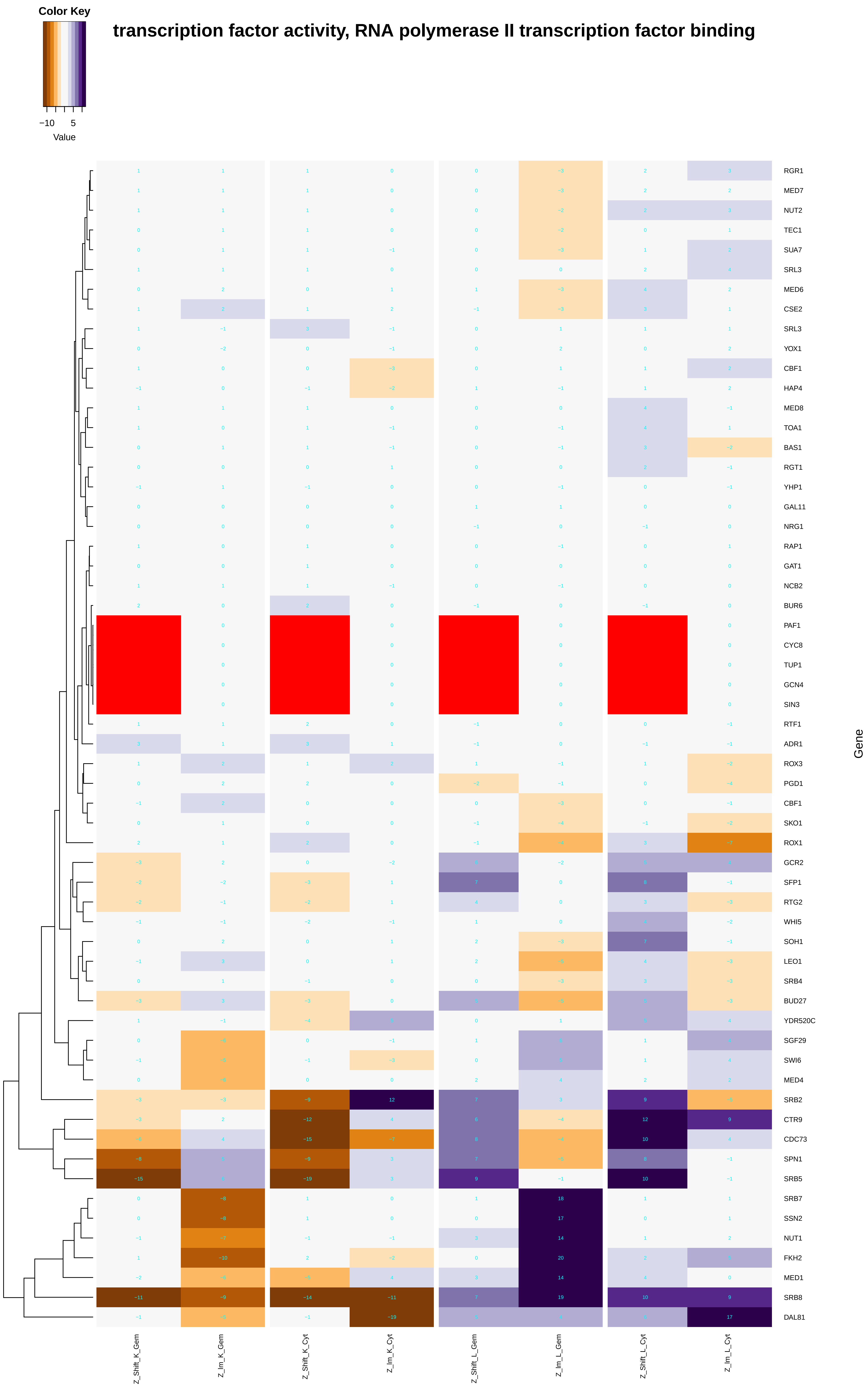
transcription factor activity, RNA polymerase II transcription factor binding
Color Key
−10
5
Value
RGR1
MED7
NUT2
TEC1
SUA7
SRL3
MED6
CSE2
SRL3
YOX1
CBF1
HAP4
MED8
TOA1
BAS1
RGT1
YHP1
GAL11
NRG1
RAP1
GAT1
NCB2
BUR6
PAF1
CYC8
TUP1
GCN4
SIN3
RTF1
ADR1
ROX3
PGD1
CBF1
SKO1
ROX1
GCR2
SFP1
RTG2
WHI5
SOH1
LEO1
SRB4
BUD27
YDR520C
SGF29
SWI6
MED4
SRB2
CTR9
CDC73
SPN1
SRB5
SRB7
SSN2
NUT1
FKH2
MED1
SRB8
DAL81
Gene
Z_Shift_K_Gem
Z_Im_K_Gem
Z_Shift_K_Cyt
Z_Im_K_Cyt
Z_Shift_L_Gem
Z_Im_L_Gem
Z_Shift_L_Cyt
Z_Im_L_Cyt

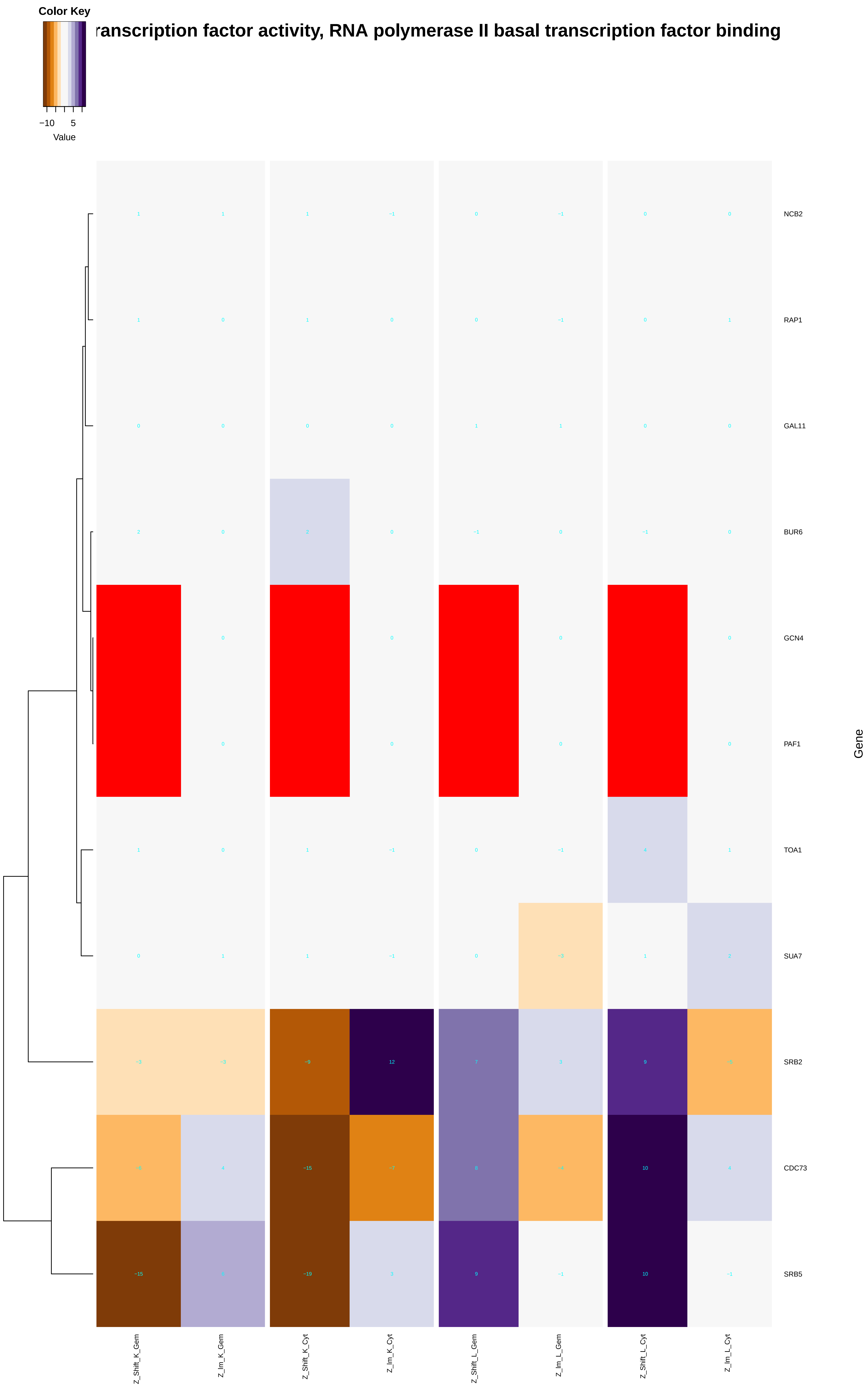

Color Key
−10
5
Value
ranscription factor activity, RNA polymerase II basal transcription factor binding
NCB2
RAP1
GAL11
BUR6
GCN4
PAF1
TOA1
SUA7
SRB2
CDC73
SRB5
Gene
Z_Shift_K_Gem
Z_Im_K_Gem
Z_Shift_K_Cyt
Z_Im_K_Cyt
Z_Shift_L_Gem
Z_Im_L_Gem
Z_Shift_L_Cyt
Z_Im_L_Cyt

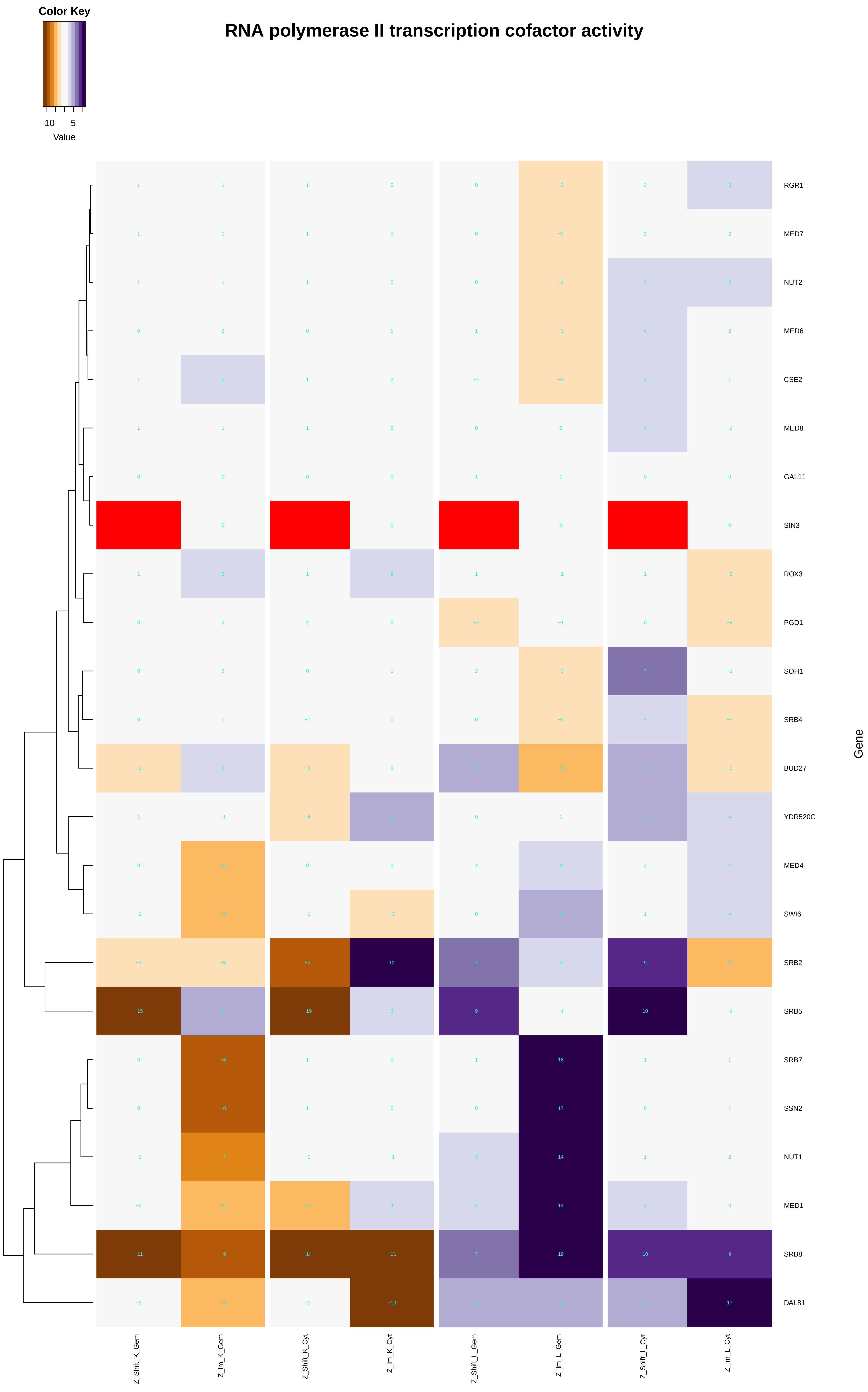

Color Key
−10
5
Value
RNA polymerase II transcription cofactor activity
RGR1
MED7
NUT2
MED6
CSE2
MED8
GAL11
SIN3
ROX3
PGD1
SOH1
SRB4
BUD27
YDR520C
MED4
SWI6
SRB2
SRB5
SRB7
SSN2
NUT1
MED1
SRB8
DAL81
Gene
Z_Shift_K_Gem
Z_Im_K_Gem
Z_Shift_K_Cyt
Z_Im_K_Cyt
Z_Shift_L_Gem
Z_Im_L_Gem
Z_Shift_L_Cyt
Z_Im_L_Cyt

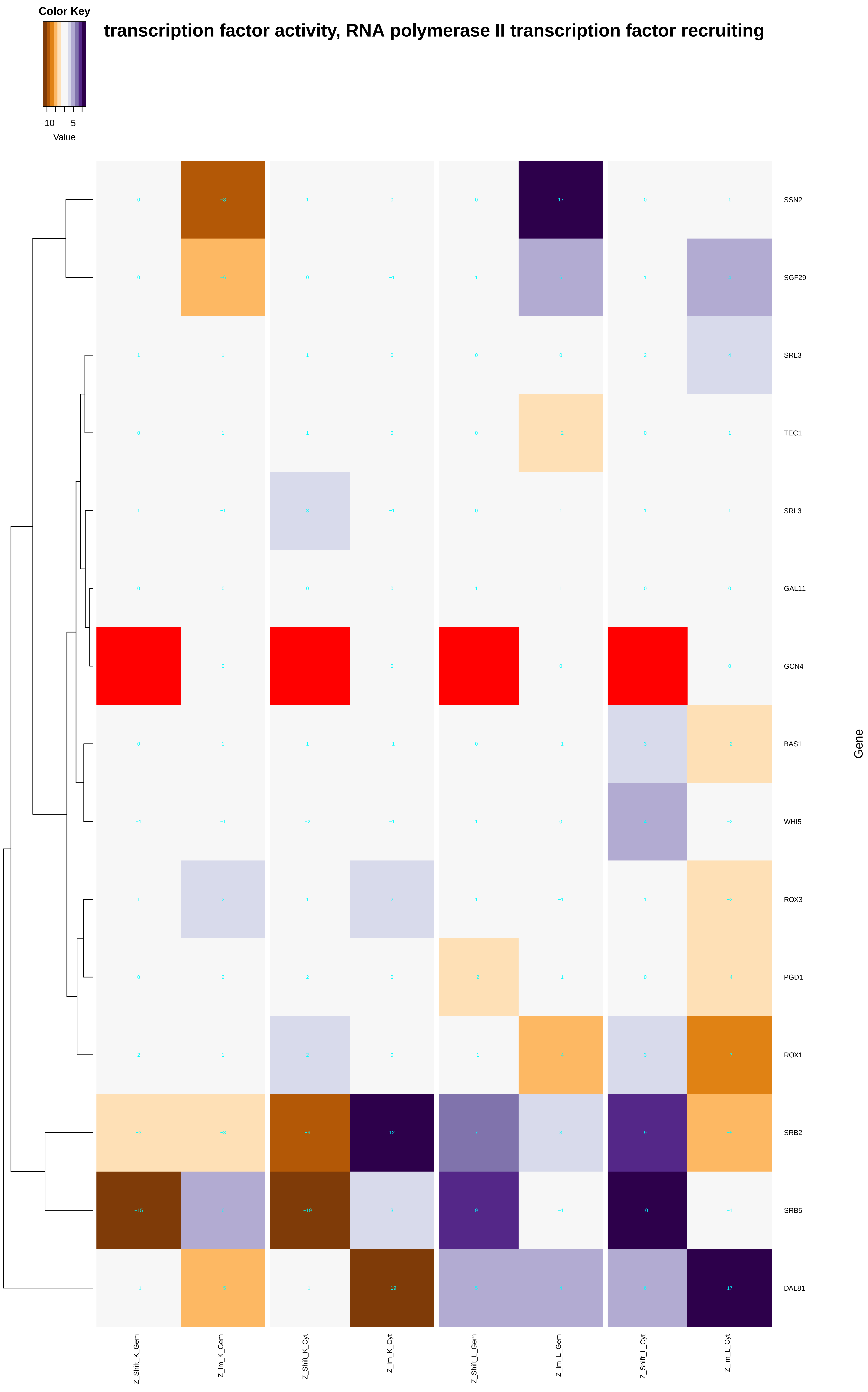
Color Key
−10 5
Value
transcription factor activity, RNA polymerase II transcription factor recruiting
SSN2
SGF29
SRL3
TEC1
SRL3
GAL11
GCN4
BAS1
WHI5
ROX3
PGD1
ROX1
SRB2
SRB5
DAL81
Gene
Z_Shift_K_Gem
Z_Im_K_Gem
Z_Shift_K_Cyt
Z_Im_K_Cyt
Z_Shift_L_Gem
Z_Im_L_Gem
Z_Shift_L_Cyt
Z_Im_L_Cyt

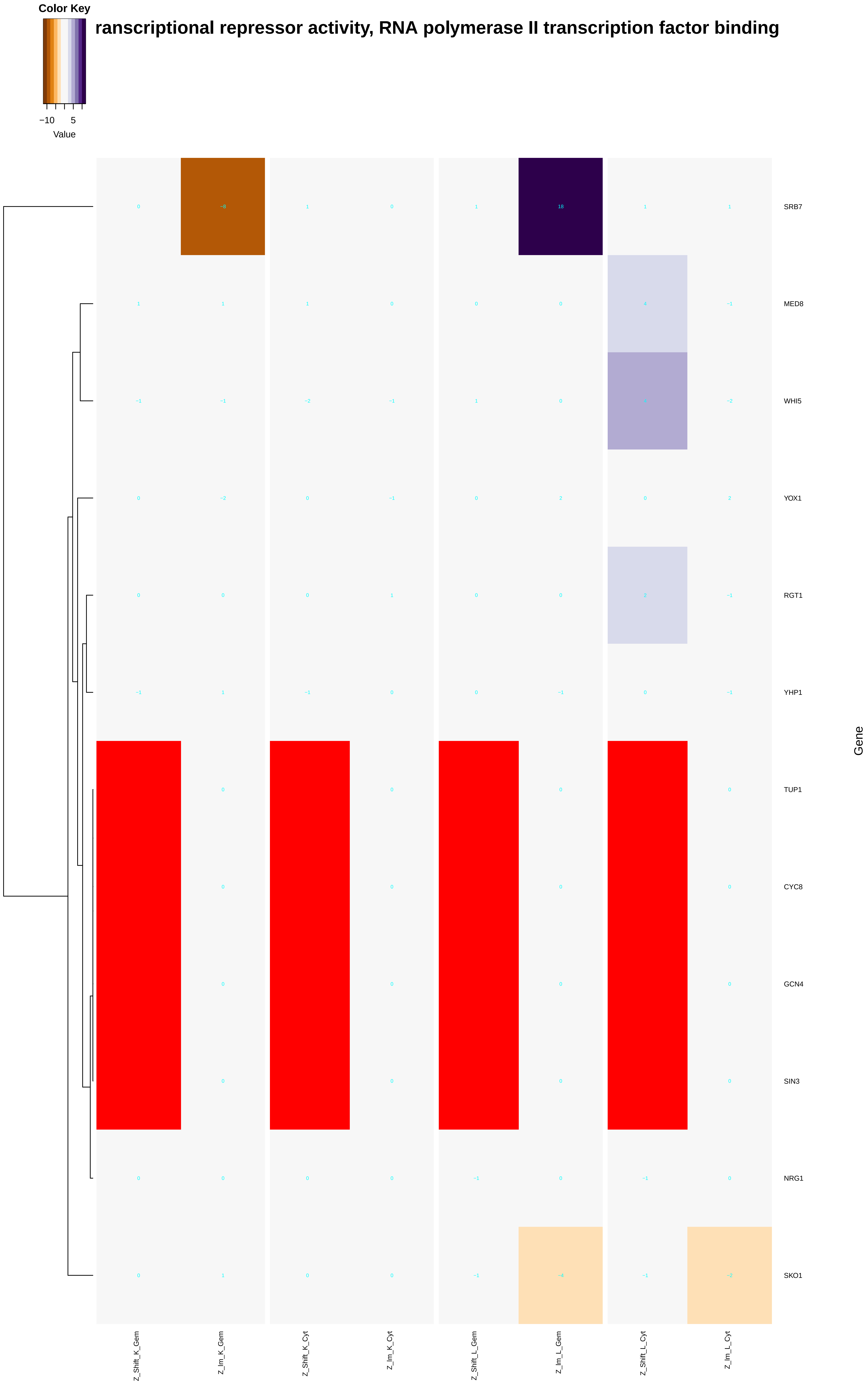

Color Key
ranscriptional repressor activity, RNA polymerase II transcription factor binding
−10
5
Value
SRB7
MED8
WHI5
YOX1
RGT1
YHP1
TUP1
CYC8
GCN4
SIN3
NRG1
SKO1
Gene
Z_Shift_K_Gem
Z_Im_K_Gem
Z_Shift_K_Cyt
Z_Im_K_Cyt
Z_Shift_L_Gem
Z_Im_L_Gem
Z_Shift_L_Cyt
Z_Im_L_Cyt

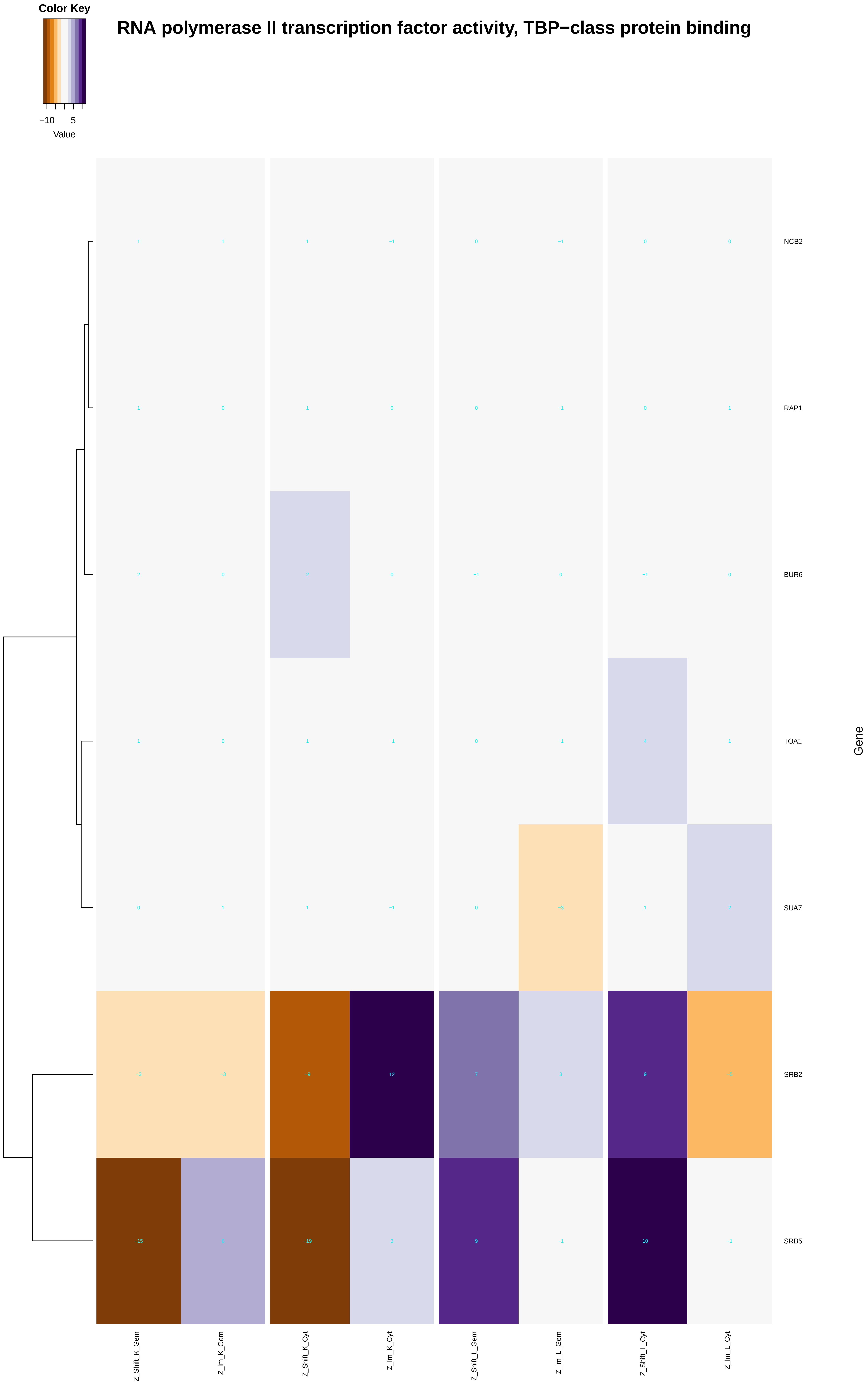
RNA polymerase II transcription factor activity, TBP−class protein binding
Color Key
−10
5
Value
NCB2
RAP1
BUR6
TOA1
SUA7
SRB2
SRB5
Gene
Z_Shift_K_Gem
Z_Im_K_Gem
Z_Shift_K_Cyt
Z_Im_K_Cyt
Z_Shift_L_Gem
Z_Im_L_Gem
Z_Shift_L_Cyt
Z_Im_L_Cyt

# RNA polymerase II transcription coactivator activity

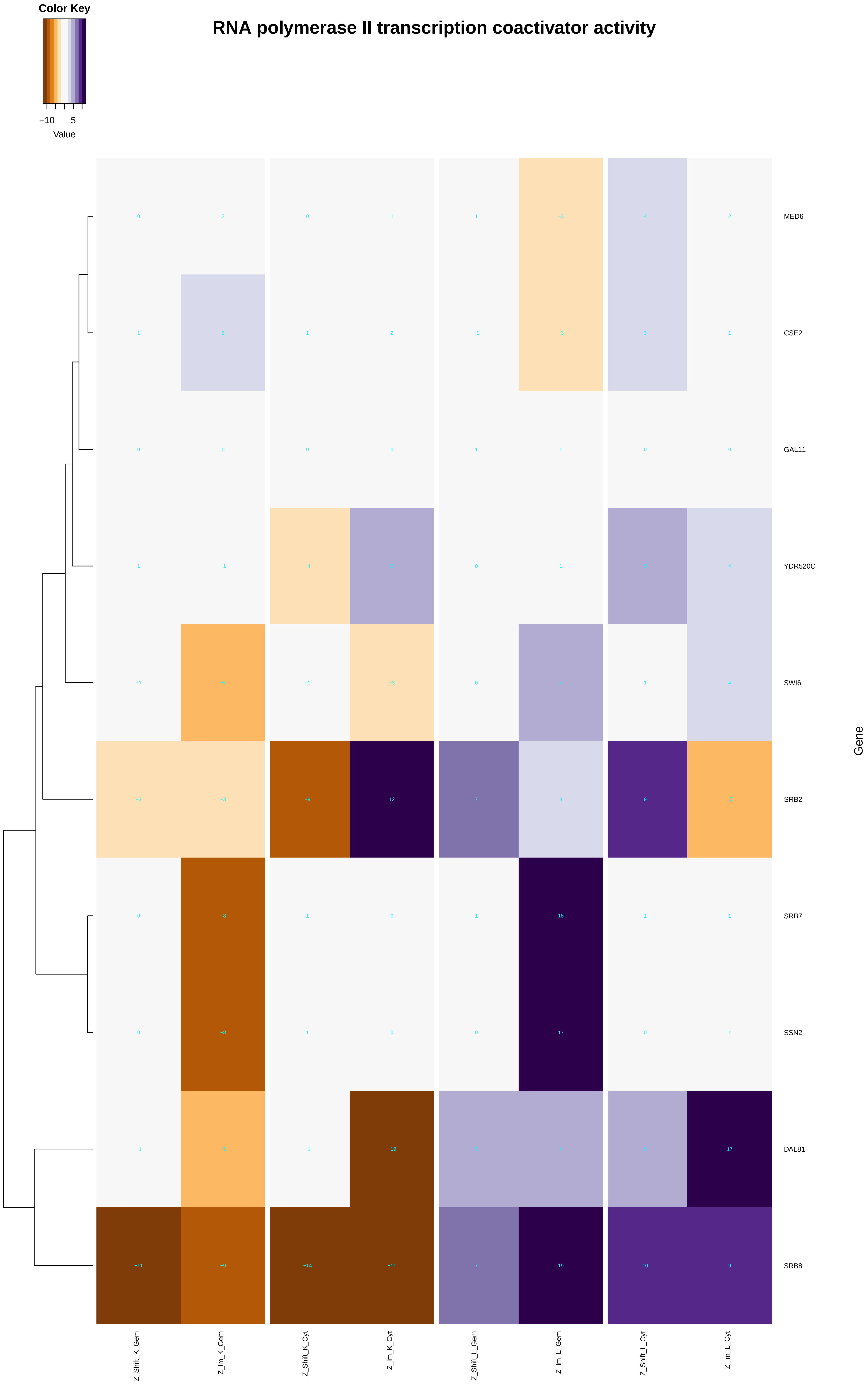

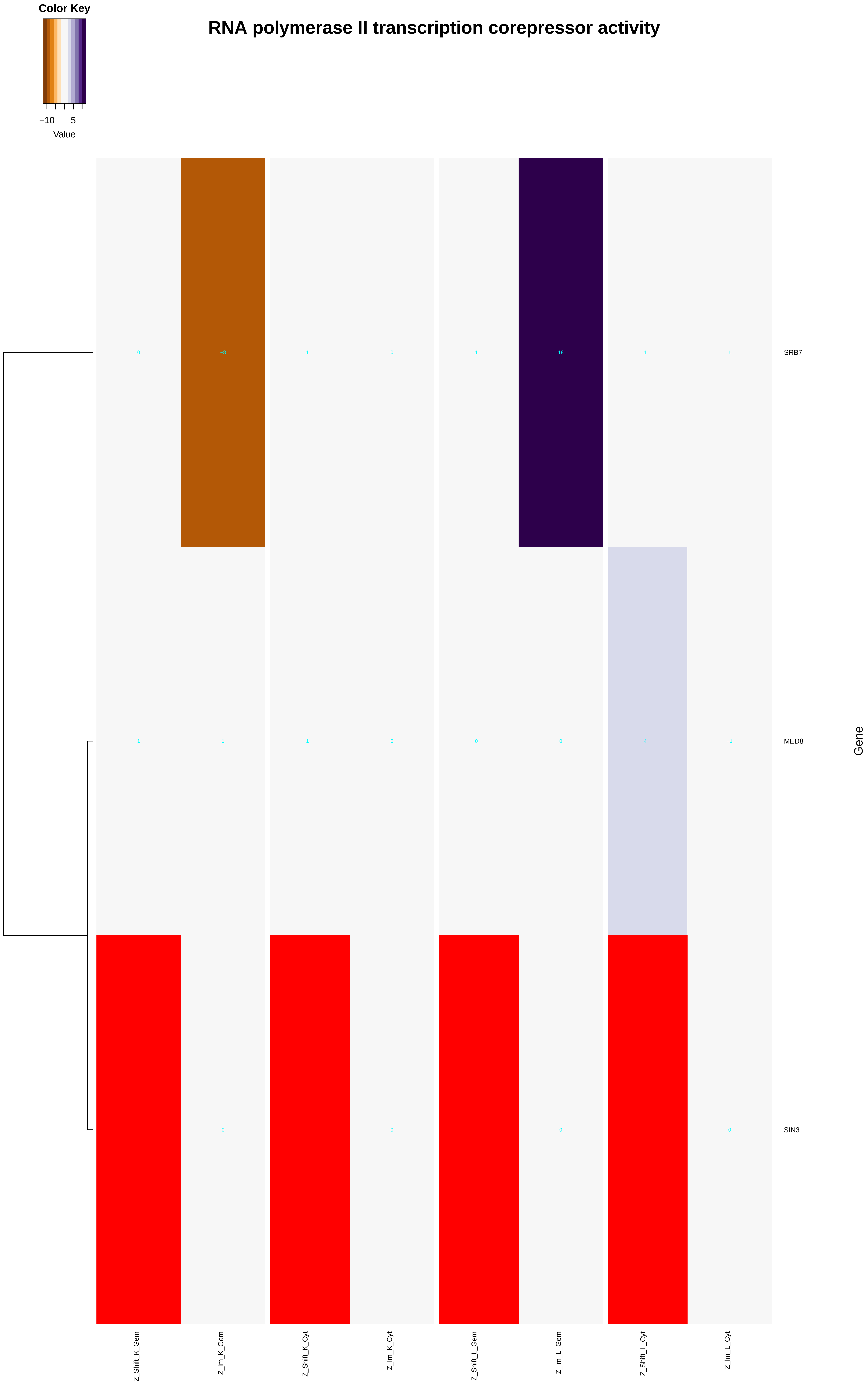

RNA polymerase II transcription corepressor activity
Color Key
−10
5
Value
SRB7
MED8
SIN3
Gene
0
−8
1
0
1
18
1
1
1
1
1
0
0
0
4
−1
0
0
0
0
Z_Shift_K_Gem
Z_Im_K_Gem
Z_Shift_K_Cyt
Z_Im_K_Cyt
Z_Shift_L_Gem
Z_Im_L_Gem
Z_Shift_L_Cyt
Z_Im_L_Cyt

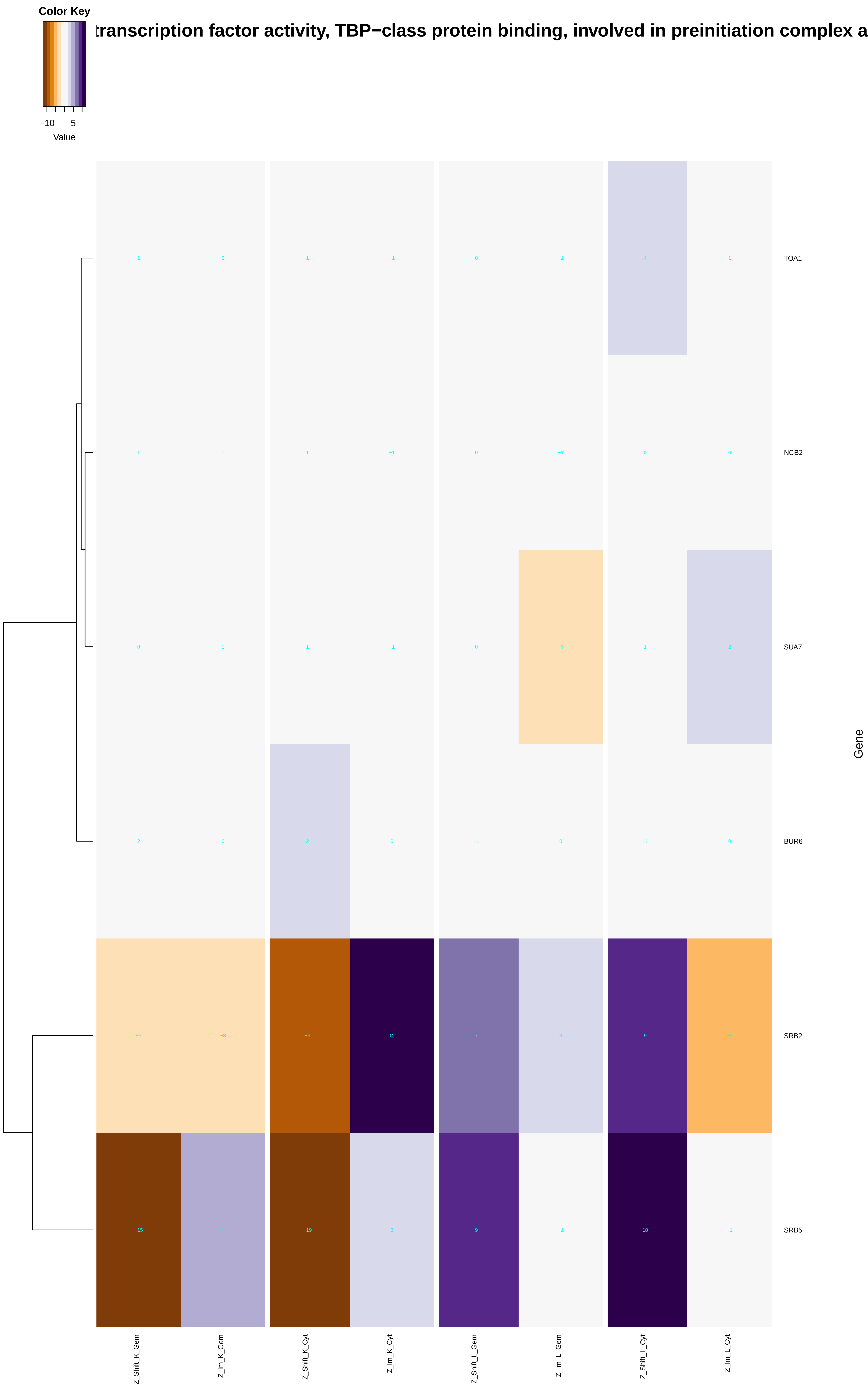
Color Key
−10
5
Value
transcription factor activity, TBP−class protein binding, involved in preinitiation complex a
TOA1
NCB2
SUA7
BUR6
SRB2
SRB5
Gene
Z_Shift_K_Gem
Z_Im_K_Gem
Z_Shift_K_Cyt
Z_Im_K_Cyt
Z_Shift_L_Gem
Z_Im_L_Gem
Z_Shift_L_Cyt
Z_Im_L_Cyt

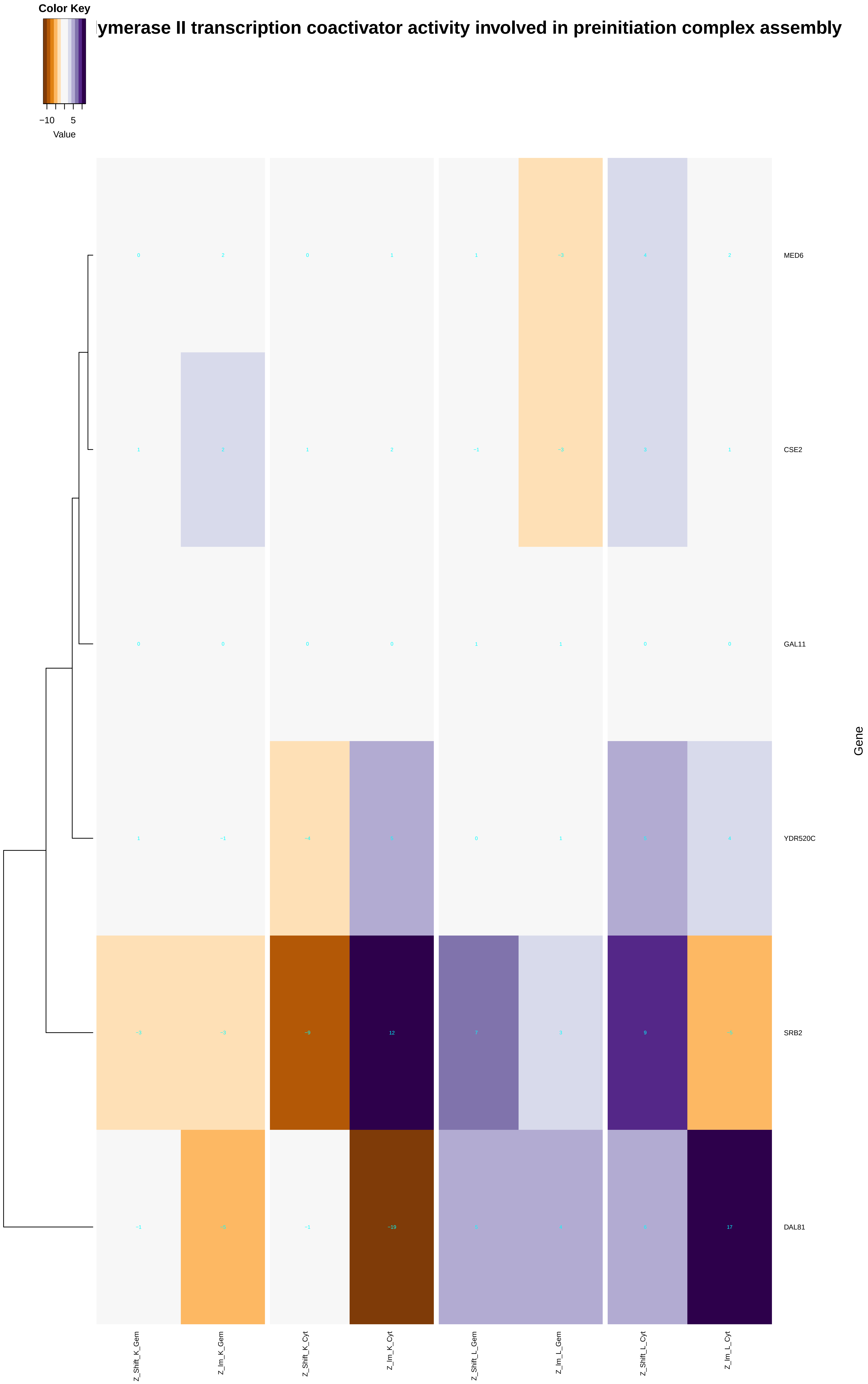

Color Key
lymerase II transcription coactivator activity involved in preinitiation complex assembly
−10
5
Value
MED6
CSE2
GAL11
YDR520C
SRB2
DAL81
Gene
Z_Shift_K_Gem
Z_Im_K_Gem
Z_Shift_K_Cyt
Z_Im_K_Cyt
Z_Shift_L_Gem
Z_Im_L_Gem
Z_Shift_L_Cyt
Z_Im_L_Cyt

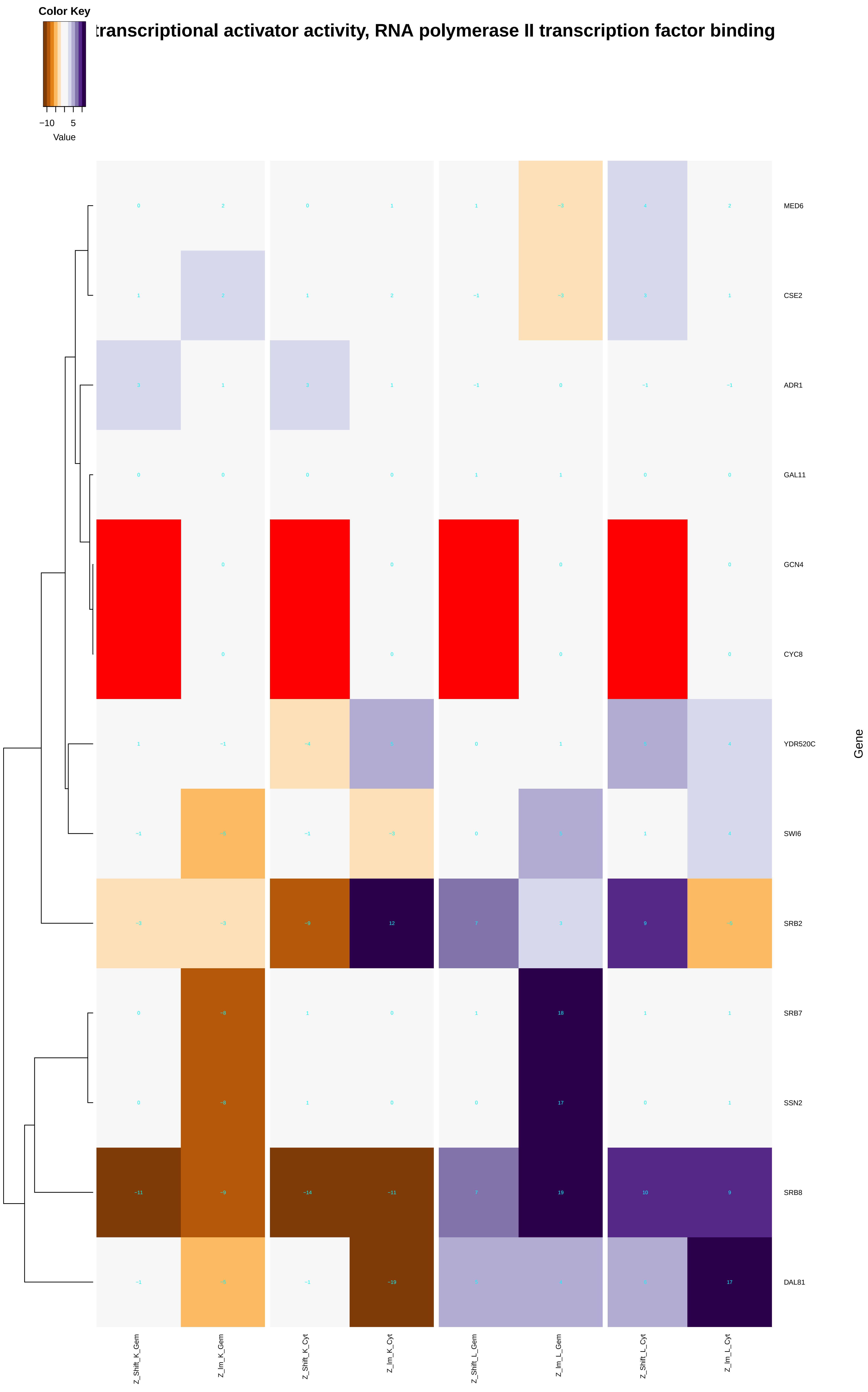
transcriptional activator activity, RNA polymerase II transcription factor binding
Color Key
−10
5
Value
MED6
CSE2
ADR1
GAL11
GCN4
CYC8
YDR520C
SWI6
SRB2
SRB7
SSN2
SRB8
DAL81
Gene
Z_Shift_K_Gem
Z_Im_K_Gem
Z_Shift_K_Cyt
Z_Im_K_Cyt
Z_Shift_L_Gem
Z_Im_L_Gem
Z_Shift_L_Cyt
Z_Im_L_Cyt

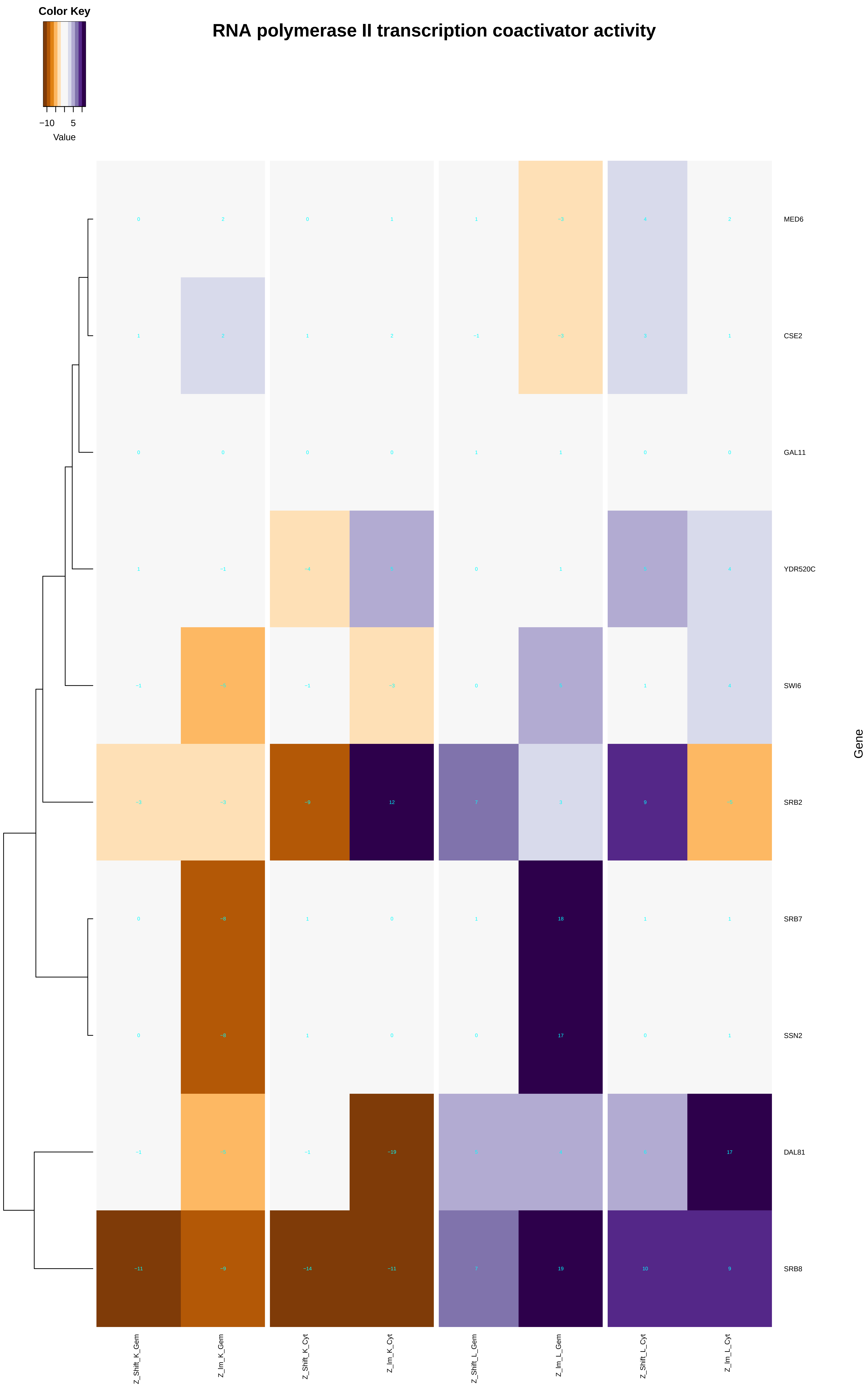
Color Key
−10
5
Value
RNA polymerase II transcription coactivator activity
MED6
CSE2
GAL11
YDR520C
SWI6
SRB2
SRB7
SSN2
DAL81
SRB8
Gene
Z_Shift_K_Gem
Z_Im_K_Gem
Z_Shift_K_Cyt
Z_Im_K_Cyt
Z_Shift_L_Gem
Z_Im_L_Gem
Z_Shift_L_Cyt
Z_Im_L_Cyt

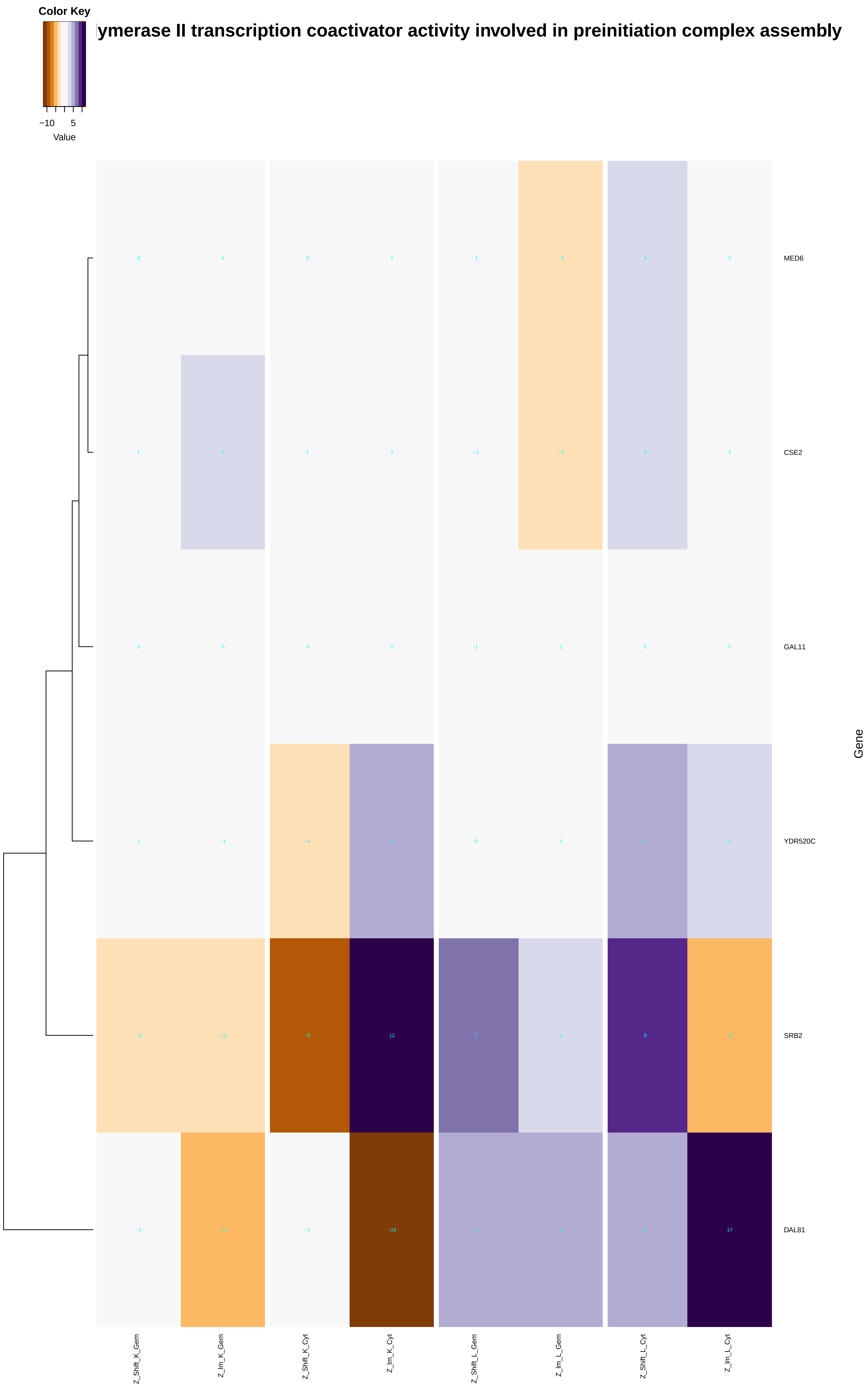

Color Key
−10 5
Value
lymerase II transcription coactivator activity involved in preinitiation complex assembly
MED6
CSE2
GAL11
YDR520C
SRB2
DAL81
Gene
Z_Shift_K_Gem
Z_Im_K_Gem
Z_Shift_K_Cyt
Z_Im_K_Cyt
Z_Shift_L_Gem
Z_Im_L_Gem
Z_Shift_L_Cyt
Z_Im_L_Cyt
